# The “speed gene” effect of *myostatin* arises in Thoroughbred horses due to a promoter proximal SINE insertion

**DOI:** 10.1371/journal.pone.0205664

**Published:** 2018-10-31

**Authors:** Mary F. Rooney, Emmeline W. Hill, Vincent P. Kelly, Richard K. Porter

**Affiliations:** 1 School of Biochemistry and Immunology, Trinity Biomedical Sciences Institute (TBSI), Trinity College Dublin, Dublin 2, Ireland; 2 UCD School of Agriculture and Food Science, University College Dublin, Belfield, Dublin 4, Ireland; Massey University, NEW ZEALAND

## Abstract

Thoroughbred horses are finely-tuned athletes with a high aerobic capacity relative to skeletal muscle mass, attributable to centuries of genetic selection for speed and stamina. Polymorphisms in the *myostatin* gene (*MSTN*), a pronounced inhibitor of skeletal muscle growth, have been shown to almost singularly account for gene-based race distance aptitude in racehorses. In Thoroughbreds, two *MSTN* polymorphisms, a single nucleotide variation in the first intron (SNP g.66493737C>T) and a non-coding transposable element within the promoter region (a 227 bp SINE insertion) are of particular interest. Until now, it has not been clear which of these variants affect skeletal muscle phenotypes or whether both can impact racing performance. In a large cohort of Thoroughbreds, we observed a complete concordance between the SNP and the SINE insertion. By means of *in vitro* assays in C_2_C_12_ myoblasts, we isolated the SNP variant from the SINE polymorphism and showed the latter is exclusively responsible for adversely affecting transcription initiation and gene expression thereby limiting myostatin protein production. Mapping the *MSTN* transcription start site in horse skeletal muscle likewise revealed anomalous transcription initiation in the presence of the SINE insertion. Our data provides mechanistic evidence that the SINE insertion uniquely accounts for the *MSTN* “speed gene” effect on race distance aptitude in the Thoroughbred horse.

## Introduction

The Thoroughbred is a domestic horse breed renowned for its athletic ability and speed which surpasses many other domestic horse breeds. Thoroughbreds originate from a narrow genetic pool that can be traced back to a limited number of Arab, Barb and Turk stallions and native mares from Britain, approximately 300 years ago [1–5]. It is well established that the athletic phenotype of the Thoroughbred is significantly influenced by their environment and training. Nonetheless, it has also been known for some decades that genetically inherited factors influence the athletic phenotype [6].

Myostatin is a member of the transforming growth factor β (TGFβ) superfamily of proteins—originally referred to as growth differentiating factor 8 (GDF-8)—that was first discovered in mice but later found to be highly conserved across species [7]. Knockout mice for the *myostatin* gene (*MSTN*) provided functional evidence that the protein is a negative regulator of skeletal muscle mass. Since then, numerous mutations in the *MSTN* gene have been reported to cause a similar phenotype in a range of species, including human [8], cattle [9–11], sheep [12], pig [13,14], goat [15] and horse [16–18]. In the dog, a heterozygote advantage in racing performance has been reported [19].

In the horse, it is well established that *MSTN* is a major quantitative trait locus that is the single most influential gene on distance aptitude [17]. Two *MSTN* polymorphisms, that are located 1,589 nucleotides apart, a SNP g.66493737C>T in the first intron (hereafter ‘SNP’) and a 227 bp SINE insertion positioned upstream of exon 1 (hereafter ‘SINE insertion’), are of particular relevance. Studies on Thoroughbreds grouped according to best race distance (either short ≤ 1,600 m or middle-long > 1,600 m) revealed a highly significant association with SNP g.66493737C>T. Horses of the CC genotype are most suited to fast, short distance racing, those with the CT genotype are most suited to middle-distance races and those with the TT genotype are more suited to longer races which require stamina. A subsequent genome-wide association study (GWAS) found a SNP on chromosome 18 (ECA18) (BIEC2-417495) to be the most significant SNP on the Illumina EquineSNP50 genotyping array when using best race distance as a quantitative phenotype [20]. This SNP and the other highest ranked SNPs in the GWAS were included in a haplotype that contained the *MSTN* gene. Re-sequencing of the flanking regions of the *MSTN* gene, identified a short interspersed nuclear element (SINE) insertion polymorphism in the region upstream of *MSTN* exon 1 that was also highly associated with the distance trait. The SINE insertion is 227 bp in length and is an equine repetitive element (ERE)-1 [21,22]. Several independent studies [23–25] have shown an association between the SNP and the SINE insertion with best race distance, supporting the findings described by Hill *et al*. [17,20]. In this context, *myostatin* in the Thoroughbred has become known as the “speed gene” [26] and is now widely used as a predictor of optimum race distance in Thoroughbreds [27,28].

In Thoroughbreds the T-allele is the ancestral ‘wild-type’ variant [29], whereas the C-allele appears to have occurred more recently, its presence becoming more prominent at the turn of the twentieth century due to changes in racing preferences towards shorter distance races and greater selection for speed over stamina traits in the Thoroughbred [29,30]. The SINE insertion arose upon the genetic background containing the C-allele [30]. The frequencies of the *MSTN* SNP C-allele and SINE insertion are moderate to high in the Thoroughbred and according to previously published data vary among populations depending on geographic region [22,29,30]. The SNP is also observed in a range of non-Thoroughbred equine breeds; however, many do not have the SINE insertion at all or it is at a very low frequency. The associations with these polymorphisms have generally been assessed separately from each other, however high linkage disequilibrium (LD) between the two has been observed in Thoroughbred horses [27,31].

In a functional context the significant associations of the *MSTN* SNP and SINE insertion with muscle fibre proportions [30–32], have suggested that the SINE insertion may be driving selection for sprinting in the Thoroughbred. Recently, using a reporter gene assay for the two variants of the *MSTN* promoter region, Santagostino *et al*. [22] showed that the presence of the SINE insertion altered myostatin protein expression. The effect of the SINE insertion was analysed following transfection of a reporter gene construct into HeLa cells (an immortal human cervical cancer cell line) and a significant decrease in *MSTN* gene expression and protein production was observed [22]. While this study suggests that the SINE insertion decreases myostatin protein levels in skeletal muscle which in turn may modify muscle development, the individual or combinatorial effect of the SNP was not considered.

The SNP is widely used commercially as a predictor of optimum race distance in the Thoroughbred, however a mechanistic relationship to racing aptitude has not been determined. Our objective was to elucidate the relative contribution that the SNP and the SINE insertion make to *myostatin* gene expression using a combination of molecular biology cell based assays and skeletal muscle biopsies from Thoroughbred horses. Our data shows that the SINE insertion underpins the mechanistic explanation of why the SNP is an appropriate predictor of best race distance in the Thoroughbred.

## Materials and methods

### Animals, ethics and licencing

Thoroughbred horses were maintained in the same training yard under the supervision and management of a single trainer. All procedures and veterinarians performing the procedures were approved and licenced (Ref: B100/3525) under the Department of Health/Irish Medicines Board/Health Products Regulatory Authority (HPRA), ethics approval (Ref: AREC-P-12-55) was granted by UCD Animal Research Ethics Committee and owners consent was given.

### Skeletal muscle biopsies

Skeletal muscle biopsies were taken from the middle gluteal muscle of a standing unsedated horse by a qualified and experienced veterinarian as per the technique described by Ledwith and McGowan [33]. The biopsy site was desensitised by subcutaneous injection of up to 5ml of 2% lignocaine prior to the biopsy. Biopsy samples were stored at -70°C until use.

### *MSTN* genotyping

Horses were genotyped for the *MSTN* SNP g.66493737C>T by Equinome Ltd. (now part of Plusvital Ireland Ltd.; http://www.plusvital.com/) as per the method described by Hill *et al*. [26].

Two polymerase chain reaction (PCR)-based assays were used to genotype horses for the *MSTN* SINE insertion 227 bp polymorphism using GoTaq Hot start master mix (Promega, WI) and associated reagents. Primers were used at a final concentration of 0.5 μM. The primer sequences were as follows: Primer set 1; 5'-ATC AGC TCA CCC TTG ACT GTA AC-3' (forward), 5'-TCA TCT CTC TGG ACA TCG TAC TG-3' (reverse); Primer set 2; 5'-ATC AGC TCA CCC TTG ACT GTA AC-3' (forward), 5'-GTA TTC TTC GTT GTG GGT TCC TC-3' (reverse). Both assays were run simultaneously using the same PCR conditions, as follows; initial denaturation at 95°C for 5 minutes, followed by 40 cycles of 95°C for 30 seconds, 58°C for 30 seconds and 72°C for 1 minute, followed by a final elongation step of 72°C for 5 minutes. The resulting amplification products were electrophoresed on a 2% agarose gel to determine the genotype of the animals.

### Gene expression

Total RNA was isolated from skeletal muscle tissue samples using Qiazol reagent (Qiagen) and homogenisation using 1.5 mm stainless steel beads in a Tissue Lyser II machine (Qiagen). RNA was isolated from C_2_C_12_ cells grown in culture using Qiazol reagent (Qiagen), scraped from wells and pipetted into microcentrifuge tubes. RNA was isolated and purified using RNeasy Plus Universal kit (Qiagen) as per the manufacturer’s instructions. Equal amounts of RNA were reverse transcribed into cDNA using the High-Capacity cDNA Reverse Transcription Kit (Applied Biosystems) as per manufacturer’s instructions. The resulting cDNA was diluted with nuclease free water and used for real-time qPCR. Specific primers were designed with the aid of Primer3Plus [34], spanning exon:exon junctions where possible. All primers were commercially synthesised (Eurofins Genomics). Sequence homology to other genomic regions was assessed using the National Center for Biotechnology Information BLAST function [35]. Hypoxanthine guanine phosphoribosyl transferase (*HPRT*) mRNA expression was used as an internal normalization control for each sample, as it was reported to be the most stably expressed gene when a panel of potential reference genes were screened in horses [36]. Careful attention was paid to avoid PCR contamination and no false-positives were observed in negative controls. Biosystems 7500 Fast Real-Time PCR System and SYBR green reagents were used to measure mRNA levels in equine skeletal muscle tissue and C_2_C_12_ cells. PCR conditions were as follows: initial denaturation at 95°C for 10 minutes, followed by 40 cycles of 95°C for 30 seconds and 58°C for 45 seconds. SDS 1.9.1 software (Applied Biosystems) was used to analyse the amplification curves and these curves were used to determine the relative mRNA expression of each gene. The expression of each gene was normalised to the expression of *HPRT* using the ΔΔCt method. The primers were as follows: *MSTN*: 5'-TTC CCA AGA CCA GGA GAA GA-3' (forward), 5'-CAG CAT CGA GAT TCT GTG GA-3' (reverse); *HPRT*: 5'-AAT TAT GGA CAG GAC TGA ACG G-3' (forward), 5'-ATA ATC CAG CAG GTC AGC AAA G-3' (reverse); *MSTN-luciferase*: 5'-CCA AGA CCA GGA GAA GAT GG-3' (forward), 5'-CAC GAT GTT GAA GTC TTC GTT G-3' (reverse); *HPRT* (mouse): 5'-AGA CTG AAG AGC TAC TGT AAT GAT C-3' (forward), 5'-CAG TGT CAA TTA TAT CTT CAA CAA TC-3' (reverse).

### Plasmid vectors

The pBluescript (+/-) plasmid was purchased from Stratagene, Agilent Technologies. The pGLuc-Basic 2 vector was purchased from New England Biolabs and was modified to add a KpnI restriction site altering the amino acid sequence from glutamic acid (E), alanine (A), lysine (K) to arginine (R), tyrosine (Y), arginine (R). This sequence incorporated the necessary KpnI recognition sequence and additionally removed the signal sequence of *Gaussia* luciferase. Modifications were made by site directed mutagenesis. The pRACE vector used for In-Fusion cloning during the experimental determination of the transcription start site of *MSTN* was purchased from TaKaRa Clontech.

### Isolation and purification of DNA for cloning of *MSTN* gene

DNA was extracted from a single equine skeletal muscle tissue sample using the RecoverEase DNA isolation kit (Stratagene, Agilent Technologies). The sample was homozygous for the SINE insertion 227 bp polymorphism and the C-allele of SNP g.66493737C>T. DNA was digested at 37°C for 2 hours with restriction enzyme NotI. NotI is a rare cutter and by *in silico* digestions it was observed that NotI did not cut within a region > 100,000 nucleotides either side of the equine *MSTN* gene. The digested DNA was purified using a QIAquick PCR purification kit (Qiagen) as per manufacturer’s instructions and eluted in nuclease free water. The *MSTN* gene was isolated and amplified by polymerase chain reaction using a TaKaRa LA PCR kit (LA PCR kit Version 2.1, TaKaRa-Clontech). The amplification primers used were 5'-CTA AAT CTG ACT CCT CTG AGA ACT G-3' (forward) and 5'-CAC ACT CTC CAG AGC AGT AAT TGG C-3' (reverse), to amplify a region of 8359 bp which included 3539 bp of the upstream promoter and 4812 bp of the *MSTN* gene. PCR was performed as follows: 94°C for 1 minute, 30 cycles of 98°C for 10 seconds and 68°C for 6 minutes, this was followed by 10 minutes at 72°C. The PCR product was purified using a QIAquick PCR purification kit as per manufacturer’s instructions and eluted in nuclease free water. XhoI and KpnI restriction enzyme recognition sequences were added to the 5 prime and 3 prime ends, respectively, of the *MSTN* gene using polymerase chain reaction. The TaKaRa LA PCR kit was used along with the following primers: 5'-GCG CTC GAG CCA GTC AAG CTC TCC AGT CAA CTA G-3' (forward) and 5'-CTC GGT ACC TGG AGT GCT CAT CAC AGT CAA GTC-3' (reverse). PCR was performed as follows: 94°C for 1 minute, 30 cycles of 98°C for 10 seconds and 68°C for 6 minutes, this was followed by 10 minutes at 72°C. The resulting PCR product was purified using the QIAquick PCR purification kit as per manufacturer’s instructions and eluted in nuclease free water. DNA was digested by the appropriate restriction enzymes and DNA was separated on an agarose gel. The gel was electrophoresed to separate DNA fragments enough to visualise and cut out easily. Extraction of DNA from the gel was performed using a QIAquick Gel Extraction kit (Qiagen) as per manufacturers’ instructions.

### Ligation and cloning of DNA

Plasmids and DNA fragments were cut with identical restriction enzymes in separate reactions prior to ligation. For insertion of the *MSTN* fragment into pBluescript or GLuc vectors the enzymes used were XhoI and KpnI and the digestions were performed as follows: Plasmids and DNA fragments were first digested with KpnI (3–5 μl DNA, 3 μl compatible Buffer, 2 μl restriction enzyme KpnI and nuclease free water to a final volume of 20 μl) at 37°C for 2 hours. These digests were then purified using the QIAquick PCR purification kit and eluted in nuclease free water, before commencing the second restriction enzyme digest. The entire purified KpnI digested reaction was then digested with XhoI (28 μl DNA, 3.5 μl compatible buffer, 2 μl XhoI enzyme and nuclease free water to a final volume of 35 μl) for 2 hours at 37°C. Subsequently, the plasmid was dephosphorylated by incubation with bacterial alkaline phosphatase (TaKaRa-Clontech) at 55°C for 2 hours. The final plasmid and DNA fragments were purified using the QIAquick PCR purification kit and eluted nuclease free water. Ligation of DNA insert and plasmid was performed using Solution I ligase from the TaKaRa DNA ligation kit (TaKaRa-Clontech). The ligation reaction was incubated at 16°C overnight (16–18 hours). After ligation, the reaction mixture was transformed into competent bacterial cells and screening by PCR and sequencing was performed to ensure correct ligation and/or modification of the plasmids had occurred.

### Modifications of plasmid DNA

Site-directed mutagenesis was employed to make single and multiple nucleotide changes to DNA sequences. Primers were designed to contain the desired mutations. The TaKaRa LATaq polymerase and associated products were used for the PCR reactions. The reaction tube was placed in a preheated PCR machine and the following PCR program was initiated: 95°C for 1 minute, 12 (for point mutations) or 18 (for multiple amino acid alterations) cycles of 95°C for 10 seconds and 55°C for 1 minute, followed by a final elongation at 68°C for 1 minute/kb of plasmid. Following the PCR, the product was purified using the QIAquick PCR purification kit and eluted in nuclease free water. The purified PCR product was digested with DpnI for 1 hour at 37°C to digest methylated and hemimethylated DNA and thus remove template DNA. After digestion, the sample was transformed into Stabl3 competent bacterial cells.

A sample (4 μg) of plasmid DNA (GLuc + MSTN plasmid, with SINE insertion 227 bp polymorphism and C-allele at SNP g.66493737C>T) was sent to GenScript (USA) for the SINE insertion 227 bp polymorphism sequence to be removed. The full plasmid was sequenced before and after removal of SINE insertion 227 bp polymorphism sequence to obtain full and thorough sequence data. GenScript removed the SINE insertion 227 bp polymorphism sequence by firstly digesting the plasmid with two rare enzymes (BoxI and AleI) which cut only once within the entire plasmid and *MSTN* sequence, on either side of the SINE insertion. The fragment was then amplified via GenScripts CloneEz method to obtain the desired new fragment (*i*.*e*. without the SINE insertion 227 bp polymorphism). After screening and sequence verification the fragment was cloned back into the vector backbone. Plasmid DNA was isolated and returned for use in further experiments.

### Preparation of plasmid DNA

Plasmids were grown at 37°C overnight on Luria Bertani (LB) agar plates with appropriate selection antibiotic prepared under sterile conditions. Single colonies were streaked and grown at 37°C overnight and subsequently LB broth cultures were inoculated with a sample of these re-streaked colonies to grow for isolation of DNA. A 4-ml volume of LB broth including selection antibiotic was inoculated with a selected colony or streak and was grown in a shaker incubator at 300 rpm at 37°C for 8 hours. This culture was centrifuged at 3,000 g for 5 minutes and the pellet was resuspended in 2 ml LB broth before being added to a final volume of 200 ml LB broth with selection antibiotic in a 1 L flask. This 200 ml culture was grown in a shaker incubator at 300 rpm at 37°C overnight (12–16 hours). The overnight bacterial culture was pelleted by centrifugation at 6,000 g for 15 minutes at 4°C. The plasmid DNA was then extracted using the Endofree plasmid Maxi Kit (Qiagen) as per manufacturer’s instructions. The concentration of the DNA was determined and it was subsequently stored at -20°C for use in transfections.

### Cell culture

C_2_C_12_ (ECACC (STR authenticated)) cells, an immortal mouse derived adherent muscle myoblast cell line [37,38], were maintained in Dulbecco’s Modified Eagle’s Medium (DMEM) GlutaMAX cell culture medium (Gibco) supplemented with 10% (v/v) Foetal Bovine Serum (FBS) (HyClone) and penicillin-streptomycin (50 U/ml and 50 μg/ml) (Gibco). Cells were grown at 37°C in a humidified environment containing 95% O_2_ and 5% CO_2_. Cells were passaged at least twice weekly depending on their levels of confluency using trypsin-EDTA (0.05% w/v) (Gibco) dissociation reagent. Myoblast cells differentiated into myotubes upon confluency and reduction of serum concentration (to 2% horse serum) in the media. C_2_C_12_ cells (< 10 passages) were transfected using lipofectamine 2000 transfection reagent (Invitrogen). C_2_C_12_ cells were routinely tested for mycoplasma infections as per the method described by Young *et al*. (2010) [39].

### Luciferase reporter assays

Luciferase reporter assays were performed on samples of conditioned media removed at various time points after transfection with plasmids containing a luciferase reporter construct. At the required time point a sample of the conditioned media was removed from the well and transferred to a microcentrifuge tube. *Gaussia* luciferase assays were performed using the Luciferase glow assay kits (Thermo Scientific Pierce) in white opaque 96-well plates. Measurements were performed using a Luminoskan^TM^ Ascent Microplate Luminometer (Thermo Scientific) and accompanying Ascent software. Conditioned media sample (20 μl) was added to the plate. The substrate (Coelenterazine) (50 μl, made up in assay buffer, provided) was added to the samples and the plate was mixed gently. The plates were read with a 2 second integration time, twice; immediately after addition of substrate and again 10 minutes after addition of substrate. Assays were performed in triplicate for each sample.

### Mapping of the transcription start site of *MSTN* gene–using SMARTer technology and 5'Rapid Amplification of cDNA Ends (5'RACE)

RNA was isolated as described previously. RNA was reverse transcribed to cDNA using the SMARTer RACE 5'/3' kit (TaKaRa-Clontech) which uses Switching Mechanism at the 5' end of RNA Template (SMART) technology and employs 5' Rapid Amplification of cDNA Ends (5'RACE) as per manufacturers’ instructions. The reaction mix was incubated for 90 minutes at 42°C followed by 10 minutes at 70°C in a hot-lid thermal cycler. Tricine-EDTA (10 μl) was then added to each reaction mixture and the 5'RACE ready cDNA was then either used immediately or stored at -20°C until use. The 5'RACE PCR was performed using SeqAmp DNA polymerase and associated buffer (TaKaRa-Clontech). The forward primer used in the reaction was the Universal primer mix supplied with the SMARTer RACE 5'/3' kit which binds to the universal primer binding sites incorporated during the SMARTer cDNA synthesis. The reverse primer used in the reaction was designed to specifically amplify the equine *MSTN* gene and was located within exon 1 of the *MSTN* gene. Both primer sequences contained a 15-nucleotide sequence which overlapped with a linearized vector for In-Fusion cloning of the fragment. Primers sequences were as follows: 5'-CTA ATA CGA CTC ACT ATA GGG CAA GCA GTG GTA TCA ACG CAG AGT-3' (forward), 5'GAT TAC GCC AAG CTT TCA GTT CCC GGA GTG GAG GAG CTT TGG-3' (reverse). A touchdown PCR program was employed, as follows; (A) 5 cycles of 94°C for 30 seconds and 72°C for 2 minutes, followed by (B) 5 cycles of 94°C for 30 seconds, 70°C for 30 seconds and 72°C for 2 minutes, followed by (C) 25 cycles of 94°C for 30 seconds, 68°C for 30 seconds and 72°C for 2 minutes. Once the PCR had finished a sample of the product was electrophoresed on a 1% agarose gel and imaged to determine the presence of product bands. If no bands or faint bands were apparent, the remainder of the PCR reaction, which was stored at 4°C during this time, was subjected to an additional 5 cycles of step C of the PCR program. The 5'RACE PCR products were electrophoresed on a 1% agarose gel and the DNA fragments of interest were located under UV light and were excised from the gel using a clean scalpel blade, as quickly as possible, to minimise the exposure time to the UV light and thus avoid damaging the DNA. The DNA was extracted from the gel using a NucleoSpin Gel and PCR Clean-Up Kit (TaKaRa-Clontech) as per manufacturers’ instructions. An In-Fusion HD Cloning enzyme master mix (TaKaRa-Clontech) was used along with a linearized pRACE vector (supplied with SMARTer RACE 5'/3' Kit). The In-Fusion cloning was performed as per manufacturers’ recommendations and the plasmid was transformed into Stellar^TM^ competent cells (TaKaRa-Clontech) and was grown at 37°C. Subsequently, the plasmid DNA was isolated and sequenced using M13 primers (5'-TGT AAA ACG ACG GCC AGT-3' (forward), 5'-CAG GAA ACA GCT ATG ACC-3' (reverse)) by MWG eurofins, to ascertain where the transcription start site was on the sequence, as the inserted fragment sequence would begin at the transcription start site, preceded by the universal primer sequence.

### Immunoblotting

Proteins were resolved by Sodium dodecyl sulphate-polyacrylamide gel electrophoresis (SDS-PAGE) as per the method of Laemmli *et al*. [40] and transferred to polyvinylidene difluoride (PVDF) membranes (Immobolin-P^SQ^; Sigma-Aldrich) using a semi-dry transfer system (Hoefer Inc.). Membranes were blocked by incubation in TBS-Tween (TBS supplemented with 0.1% (v/v) Tween) (TBST) supplemented with 5% (w/v) non-fat dry milk powder for 1 hour at room temperature. Blots were then incubated in polyclonal primary antibody (rabbit anti-GLuc, Catalogue no. E8023S, New England Biolabs at 1:1000) diluted in fresh blocking buffer overnight at 4°C. The secondary antibody used was an anti-rabbit horse-radish peroxidase-linked (HRP) antibody diluted in blocking buffer at a 1:5000 dilution. The secondary antibody was incubated with the membrane for 1 hour at room temperature before blots were developed using an enhanced chemiluminescence (ECL) detection system (Millipore Immobilon ECL Western Blotting Substrate) for detecting horseradish peroxidase labelled antibody, by means of the HRP catalysed oxidation of luminol under alkaline conditions and the results were visualised by ChemiDoc (Bio-Rad) computerised system and Image Lab software. Densitometry analysis was performed using Image Lab Software Analysis function.

### Statistical analysis

Statistical analyses were performed using Prism 5.0 software (GraphPad). All results are expressed as mean ± SEM unless otherwise indicated. Mean values were compared using a two-way ANOVA (means (fixed) model) (luciferase assay time-course data) or a one-way ANOVA (all other data) with a Bonferroni multiple comparison post-test with 95% confidence intervals. A *P*-value of ≤ 0.05 was taken to indicate significance, corresponding to the applied confidence interval of 95%, (* = *P* ≤ 0.05, ** = *P* ≤ 0.01, *** = *P* ≤ 0.001).

## Results

### The *myostatin* SINE insertion and SNP are in full concordance

Genotypes for the SNP were denoted as CC (homozygous C-allele), CT (heterozygous) and TT (homozygous T-allele, ‘wildtype’), whereas annotations used to distinguish the SINE insertion genotypes were II (homozygous SINE insertion), IN (heterozygous) and NN (homozygous no SINE insertion). Complete concordance between the SINE insertion and the SNP was observed (100%) for all horses genotyped for the SNP and SINE variants (*n* = 145) (Table 1).

**Table 1:** The myostatin SINE insertion and SNP are in full concordance in the Thoroughbred.

		SNP g.66493737C>T			
		CC	CT	TT	Total
SINE insertion 227 bp	II	49	0	0	49
	lN	0	54	0	54
	NN	0	0	42	42
	Total	49	54	42	145

Thoroughbred horses (*n* = 145) were genotyped for both the *MSTN* g.66493737C>T SNP and the SINE insertion 227 bp polymorphism, the table shows the numbers of horses of each combination of genotypes. SNP genotypes were denoted; CC = homozygous C-allele, CT = heterozygous, TT = homozygous T-allele and SINE insertion genotypes were denoted; II = homozygous SINE insertion, IN = heterozygous and NN = homozygous no SINE insertion.

### *MSTN* transcript levels correlate with *MSTN* genotype

**Fig 1:**
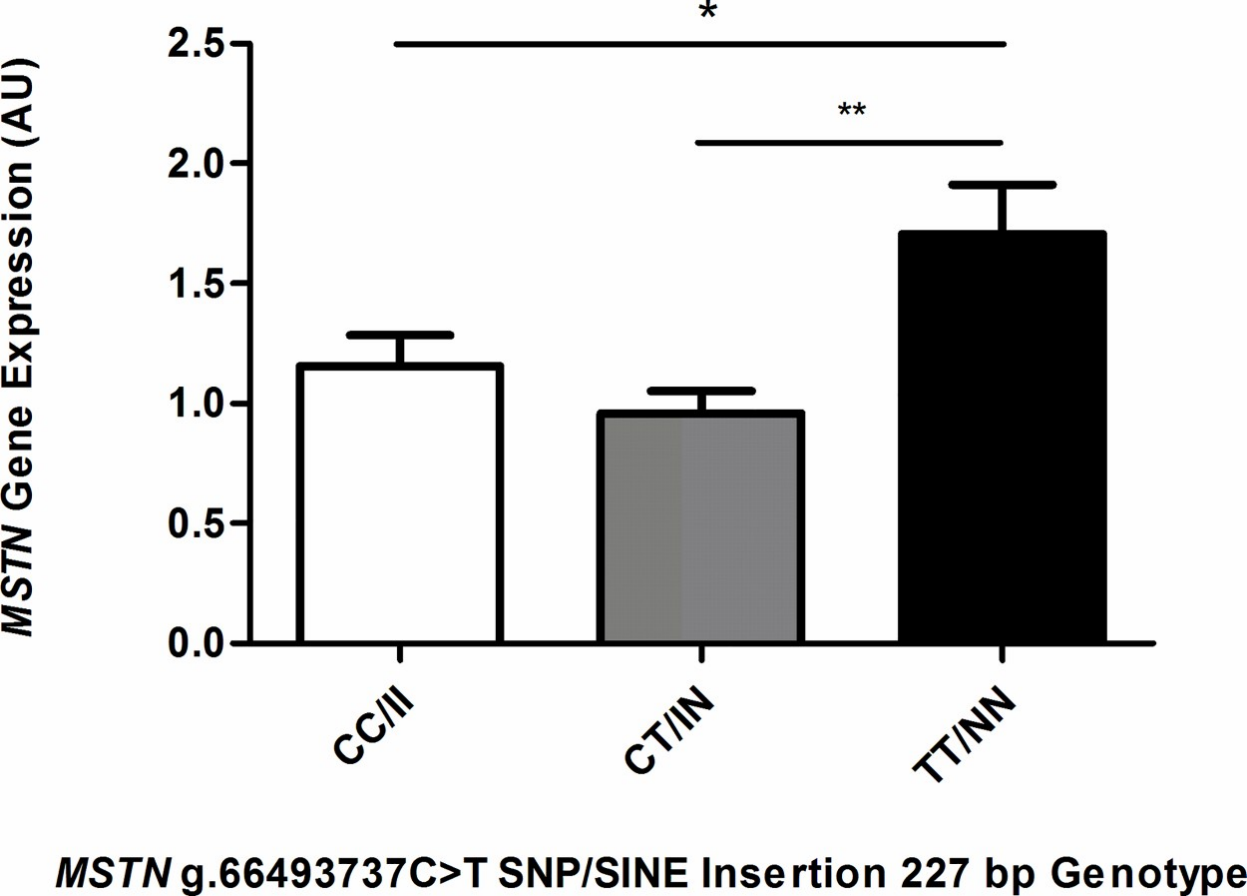
*MSTN* transcript levels correlate with *MSTN* genotype. *MSTN* gene expression levels, CC/II: *n* = 36, CT/IN: *n* = 34 and TT/NN: *n* = 11, performed in at least duplicate. Gene expression was normalised to the expression of *HPRT* (* = *P* ≤ 0.05, ** = *P* ≤ 0.01, *** = *P* ≤ 0.001).

Considering the genomic locations of the polymorphisms, we investigated their association with *MSTN* gene expression in skeletal muscle in a cohort (*n* = 81) of untrained Thoroughbred horses (21 ± 3 months). Significantly higher levels of *MSTN* mRNA transcripts were associated with the TT/NN genotype compared to CT/IN (P ≤ 0.01) and CC/II (*P* ≤ 0.05) (Fig 1). There was no observed difference in *MSTN* mRNA levels between the CC/II and the CT/IN genotypes (*P* > 0.05).

### The SINE insertion significantly affects *myostatin* gene expression and protein levels

**Fig 2:**
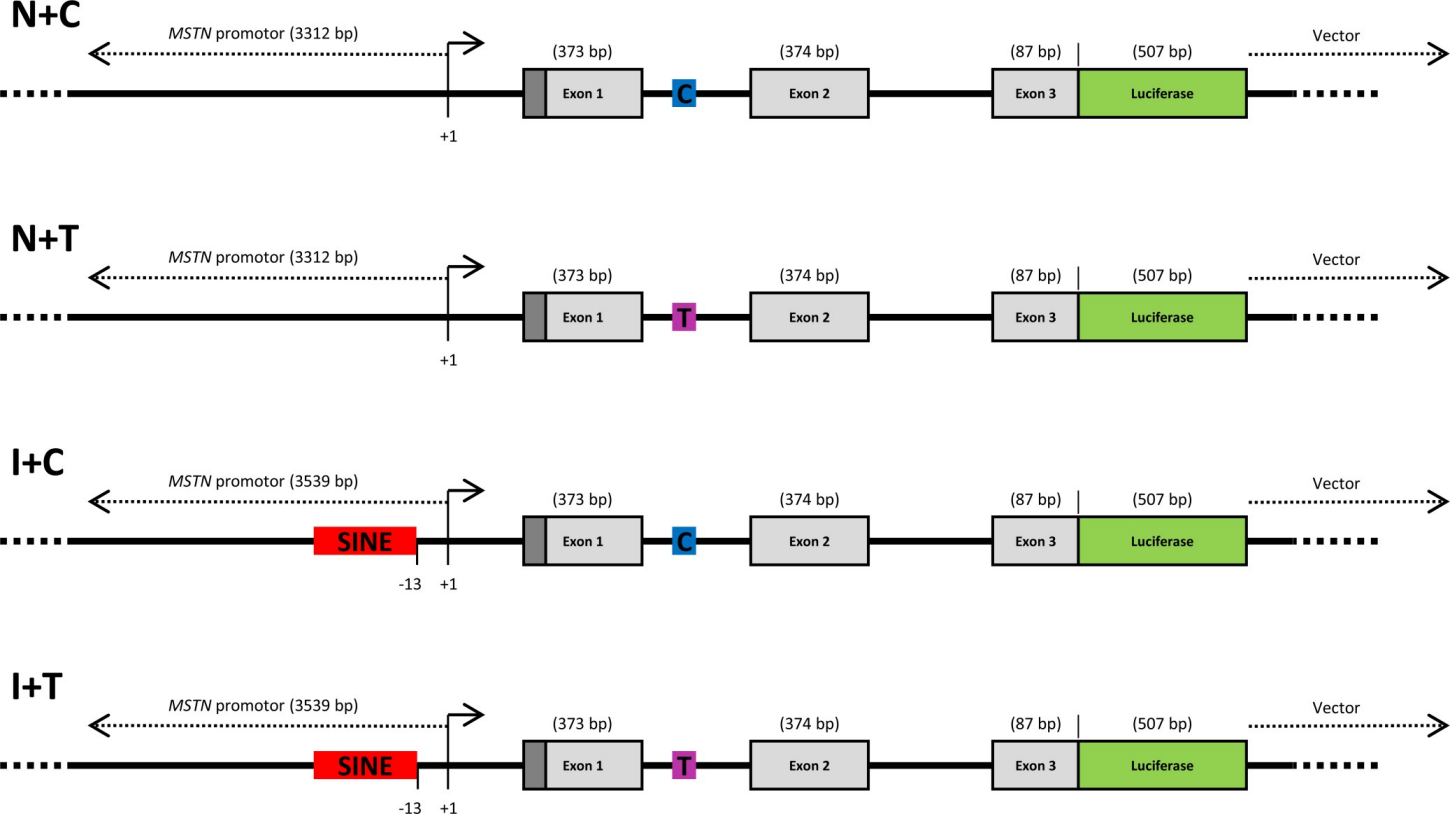
Schematic of MSTN-luciferase constructs. Figure displays four diagrams depicting linearized versions of the constructs. The four variants differ only in the presence or absence of the SINE insertion 227 bp polymorphism (RED) and the nucleotide at SNP g.66493737C>T (C = BLUE, T = PINK). The organisation of the protein is depicted also (myostatin signal sequence = DARK GREY, myostatin pro-peptide (precursor protein) = LIGHT GREY, luciferase = GREEN). Diagrams are not to scale.

To determine which of the polymorphisms was responsible for the effect on gene expression, we used a *Gaussia* luciferase reporter gene assay. Four polymorphic variants were created for the luciferase reporter assay, which differed for the SNP variants and the presence or absence of the SINE insertion. Constructs were created containing the *MSTN* promoter region (3539 bp) and the portions of the gene encoding the signal sequence and precursor protein (4812 bp) after which the cDNA sequence for *Gaussia* luciferase was inserted in place of the mature region of the myostatin protein. The inclusion of the signal sequence and precursor protein of myostatin allowed secretion of the *Gaussia* luciferase into the culture media for quantification. Linear schematics of the four constructs are displayed in Fig 2.

**Fig 3:**
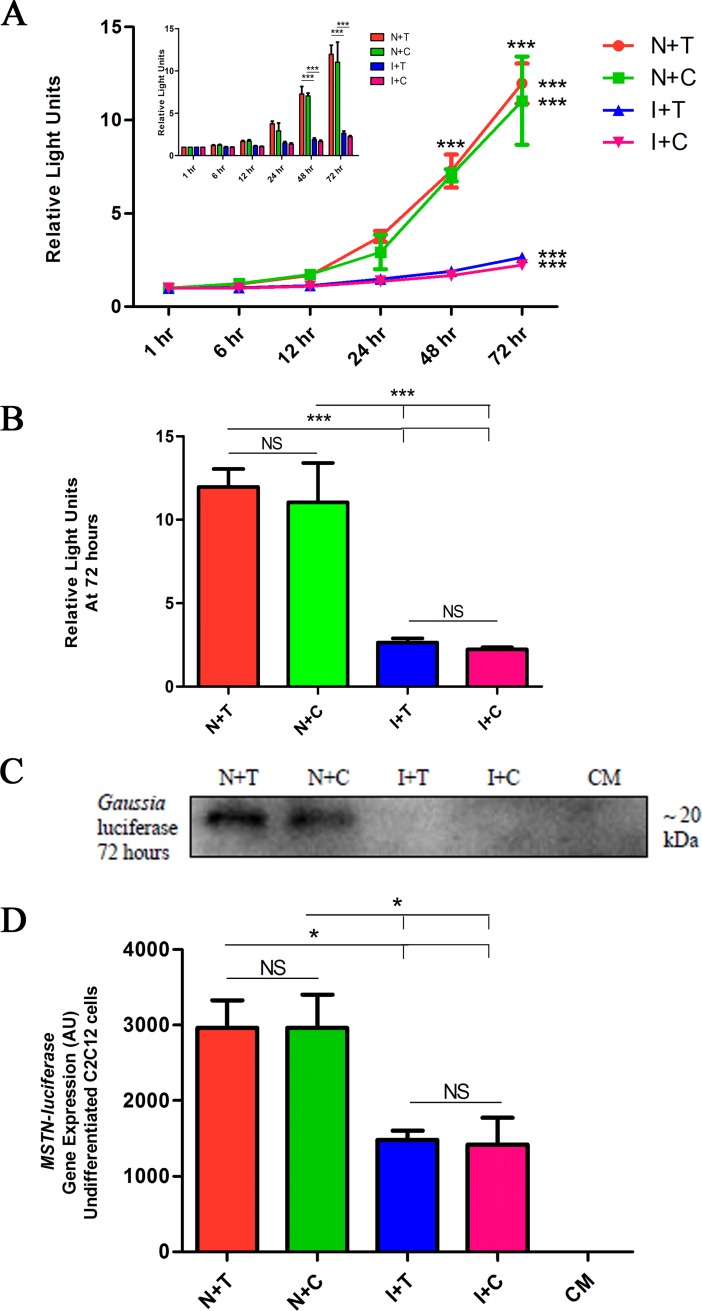
The SINE insertion affects myostatin gene expression and protein levels. C_2_C_12_ myoblasts were transfected with MSTN-luciferase plasmids, 24 hours later the media was refreshed to remove the plasmid and lipofectamine mixture. Samples of cultured media were removed at various time points and luciferase assays were performed to measure the activity and thus the amount of myostatin being produced. Activity data was normalised to the 1 hour time point. (A) *Gaussia* luciferase production as measured by luciferase assay, mean ± SEM of *n* = 3 independent experiments, performed in triplicate. *P* values where shown indicate significance, asterisk above time points indicates variance between groups, asterisk to the right of the line graph indicates variance due to time, for that group. (A-insert) Equivalent data in bar chart format. (B) Luciferase assay data at 72 hours. (C) Immunoblot for *Gaussia* luciferase in secreted media at 72 hours. CM indicates conditioned media from untransfected cells. A representative image of 3 separate experiments performed is shown. (D) Gene expression of *MSTN-luciferase* transfected undifferentiated cells C_2_C_12_ cells at 72 hours, *n* = 4 for each plasmid, performed in at least duplicate. Gene expression was normalised to the expression of *HPRT* (* = *P* ≤ 0.05, ** = *P* ≤ 0.01, *** = *P* ≤ 0.001).

The four constructs were transfected into both undifferentiated C_2_C_12_ myoblasts and differentiated C_2_C_12_ myotubes and the production of luciferase, as a proxy for myostatin production, was assayed over a 72-hour time course. Using the secreted luciferase assay, with the data normalised to the 1 hour time point, we observed a highly significant (*P* ≤ 0.001) effect of the SINE insertion on the production of luciferase (hence myostatin) in undifferentiated cells (Fig 3A). After 72-hours the presence of the SINE insertion resulted in a 4.5-fold decrease in myostatin production (Fig 3B), with the difference among genotypes being statistically significant as early as 48-hours in the undifferentiated cells (*P* ≤ 0.001). The SNP had no effect on luciferase production (*P* > 0.05). An immunoblot for the *Gaussia* luciferase protein confirmed the activity assay data (Fig 3C). Although transfection efficiency was lower in the differentiated cells (myotubes) a similar profile of luciferase production was observed (S1 Fig).

To evaluate whether the differential production of myostatin in the presence of the SINE insertion was the result of reduced *MSTN* gene transcription, we measured *MSTN* mRNA levels by the luciferase reporter transfection into C_2_C_12_ cells. The mRNA production from the MSTN-luciferase construct was significantly decreased (*P* ≤ 0.05) in the presence of the SINE insertion but was unaffected by the SNP (*P* > 0.05) in undifferentiated C_2_C_12_ cells (Fig 3D). Again, although transfection efficiency was lower in the differentiated cells a similar profile of mRNA production was observed (S1C Fig).

### *In silico* predictions suggest the SINE insertion alters the *MSTN* transcription start site

Considering the observed differences in *MSTN* gene expression arising from the SINE insertion we hypothesised that its presence may alter the recognition of the transcription start site (TSS) by RNA polymerase. Initially, this was investigated by means of an *in silico* approach.

**Fig 4:**
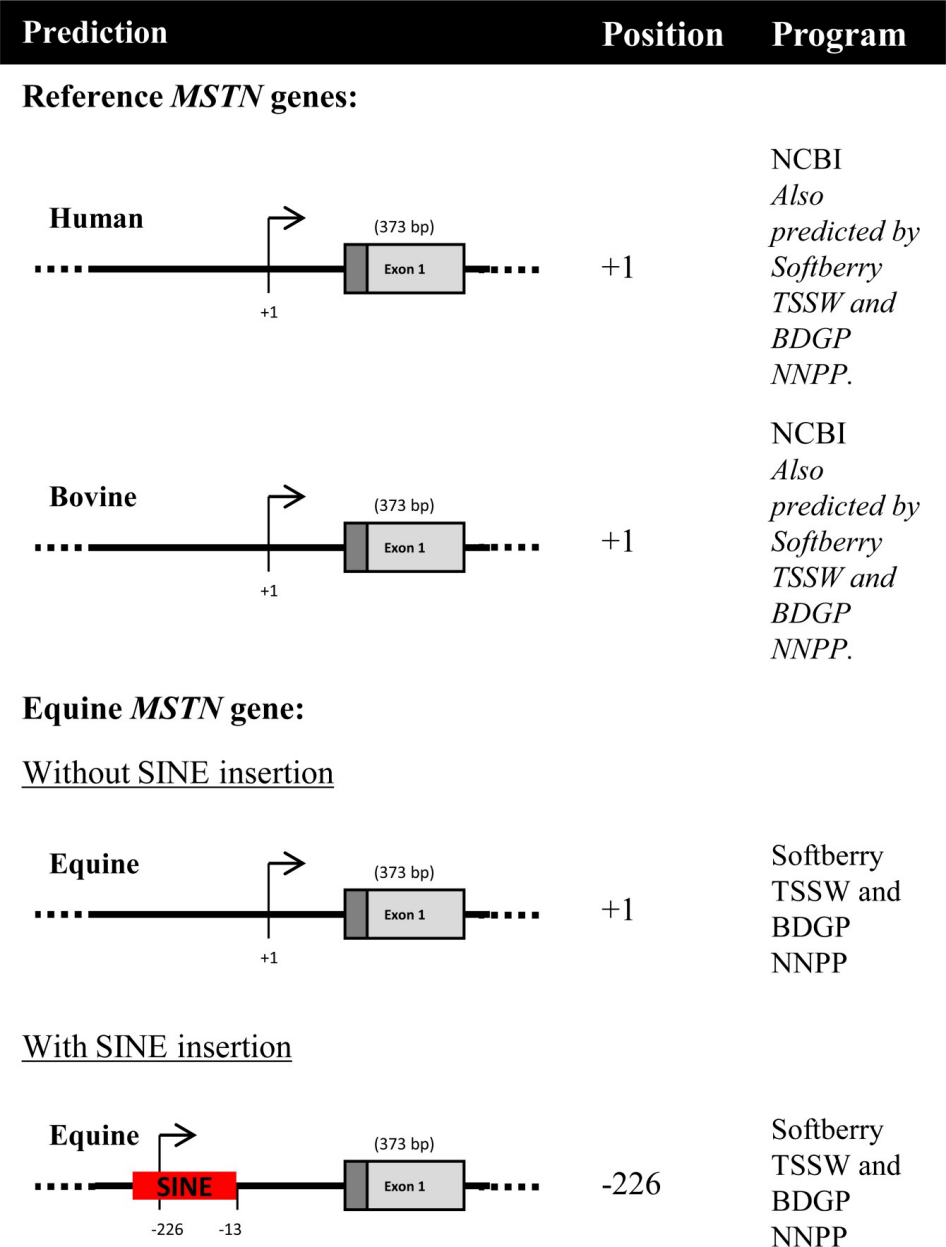
*In silico* methods predict the *MSTN* transcription start site is altered by the SINE insertion. For reference, the human and bovine *MSTN* gene TSS are depicted at the top of the figure. The human and bovine *MSTN* TSS are both in the same position, 133 bp upstream of the ATG, translation initiation site and are denoted +1. The TSS for the equine *MSTN* gene was predicted using two software packages (available online); Softberry TSSW [43] and BDGP NNPP [44–46]. The TSS for the gene without the SINE insertion was predicted to be at +1 and with the SINE insertion at -226. Positions noted for equine TSS are in relation to the human/bovine TSS at +1.

The TSS of the equine *MSTN* gene has not been experimentally determined but has been predicted [16] based on the location of the TSS in the human and bovine gene, with which it shares high sequence homology. The first nucleotide in the human *MSTN* cDNA sequence (NCBI reference sequence: NM_005259.2) has been denoted +1 [41]. The bovine *MSTN* TSS is in the same position (NCBI reference sequence: NM_001001525.3), which is 133 bp upstream of the translation initiating methionine [42]. The human and bovine *MSTN* TSS are both depicted in Fig 4 for reference.

Our *in silico* predictions of the *MSTN* TSS were obtained using Softberry TSSW [43] and BDGP Neural Network Promoter Prediction (NNPP) [44–46]. The human and bovine *MSTN* TSS were correctly predicted to be in the same position as that determined experimentally (+1); instilling confidence in the accuracy of the prediction methods.

*In silico* predictions of the equine *MSTN* TSS (Fig 4) obtained using these programs predicted the ‘wild-type’ no-SINE insertion *MSTN* TSS (+1) to be situated in the same position as that of the human and bovine *MSTN* genes. In the presence of the SINE insertion, transcription was predicted to start at -226. Furthermore, *in silico* insertion of the equine SINE insertion sequence into the human or bovine *MSTN* gene sequences at the equivalent position altered the predicted TSS to be at the same position as that of the equine *MSTN* gene, that is, within the SINE insertion (-226). Therefore, these predictions indicate a displacement of the *MSTN* TSS in the presence of the SINE insertion.

### The *MSTN* transcription start site is altered by the SINE insertion

Further to our *in silico* predictions, we experimentally determined the TSS of the *MSTN* gene in the presence (CC/II) and absence (TT/NN) of the SINE insertion. To do so, RNA was isolated from equine *gluteus medius* skeletal muscle tissue and 5’ RACE was used to recover sequence data for the 5’-end of the myostatin mRNA transcript.

**Fig 5:**
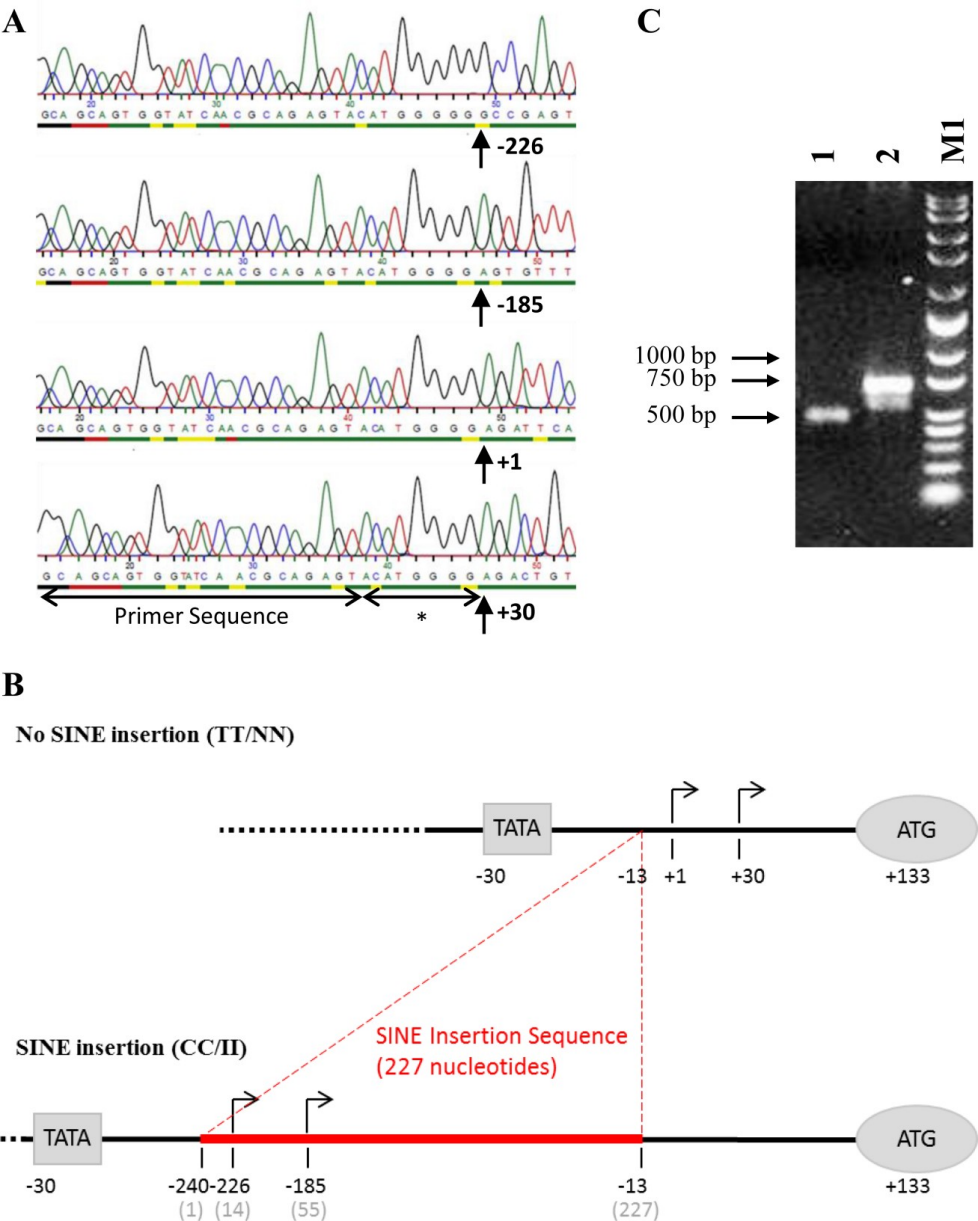
The *MSTN* transcription start site is altered by the SINE insertion. RNA was isolated from equine *gluteus medius* skeletal muscle tissue and 5'RACE was used to recover sequence data for the 5'-end of the myostatin mRNA transcript. DNA was sequenced using M13 primers by MWG eurofins. (A) Displays snapshots of the raw sequencing data obtained with transcription start sites (TSS) indicated. The SMARTer 5'RACE primer sequence is shown prior to the TSS along with 8 additional bases (*) which were added to first-strand cDNA; during reverse transcription, when the SMARTScribe reverse transcriptase reaches the 5’ end of the RNA, its terminal transferase activity adds a few additional nucleotides to the 3’ end of the first-strand cDNA. The same set of 8 bases (ACATGGGG) is observed in each clone. (B) Depiction of a non-SINE insertion *MSTN* gene (top) and a SINE insertion *MSTN* gene (bottom), with experimentally determined TSS indicated. Red lines indicate the position of the SINE insertion sequence. Numbering of nucleotide bases established from the human TSS (and the *in silico* predicted equine transcription start site) as +1. Position of the ATG (predicted translation start site) and predicted TATA box are also marked on diagram. (C) 5'RACE PCR products were electrophoresed on a 1.5% agarose gel, 1: TT/NN non-SINE insertion sample; 2: CC/II SINE insertion sample; M1: wide range MW markers (Sigma).

We found that the presence of the SINE insertion altered the transcription start site of the gene. The ‘wild-type’ no-SINE insertion displayed two putative TSS, at +1 and +30, numbered from the predicted TSS at +1. The TSS at +1 accounted for 85% of the sequenced clones. The alternate TSS, which was 30 nucleotides downstream into the predicted 5'UTR, was infrequently observed. The TSS at +1 matches that of the human and bovine *MSTN* genes and is the predicted start site according to *in silico* analysis (Fig 4). In the presence of the SINE insertion two alternative TSS were observed; –226 (14 nucleotides into the SINE insertion sequence) and –185 (55 nucleotides into the SINE insertion sequence). Notably, the ‘wild-type’ TSS (+1) was not observed in samples with the SINE insertion. Fig 5A displays the raw sequence data for each TSS and Fig 5B shows a diagrammatic representation of the positions of the observed TSS. The alteration of the transcription start site was apparent from the change in transcript length (Fig 5C) visualised following agarose gel electrophoresis. The ‘wild type’ no-SINE insertion sample produced a product approximately 200 nucleotides smaller than the SINE insertion sample.

Collectively, these data provide experimental and computational evidence that the *MSTN* TSS is altered due to the presence of the SINE insertion in equine skeletal muscle.

## Discussion

This study provides mechanistic evidence that the SINE insertion disrupts normal *MSTN* expression. The results of this study also show that the SNP and the SINE insertion are in full concordance in this population of Thoroughbreds. Together the results validate the use of the SNP as a predicative marker, enabling allocation of horses to their most suitable race distance and mate selection for breeding for specific racing traits.

Contrary to previous findings [22,27,30,47] we have found full concordance between the SNP and the SINE insertion in the Thoroughbred. We suggest that these variances likely arise from technical differences in the experimental strategy, resulting in allelic dropout during PCR amplification in earlier studies.

To elucidate the biochemical manifestations of these polymorphisms we constructed four luciferase reporter gene vectors; two created to mimic the naturally occurring genotypes (SINE insertion + C-allele and no SINE insertion + T-allele) and two created to distinguish the effects of the two polymorphisms (no SINE insertion + C-allele and SINE insertion + T-allele). By utilizing these constructs *in vitro*, we could study the effects of both polymorphisms on myostatin protein production and gene expression both individually and in combination.

*MSTN* transcript levels in muscle biopsy samples from Thoroughbred horses showed a significant (*P* ≤ 0.05) association with the *MSTN* genotype, wherein the SINE insertion/C-allele of the SNP resulted in decreased *MSTN* transcript levels. However, due to the concordance of the polymorphisms it was not possible to resolve a predominant transcription effect to either polymorphism. However, by means of *in vitro* constructs, it was possible to study the effects of the polymorphisms, individually and in combination, on myostatin protein production and gene expression. We showed that the SINE insertion alone can affect myostatin protein production, with a 4.5-fold decrease (*P* ≤ 0.001) in myostatin production. By contrast, the SNP had no measurable effect on its own nor did it aid or attenuate the effect of the SINE insertion. The results clearly confirm that the SINE insertion is the predominant functional mutation in Thoroughbreds. Furthermore, the *MSTN* transcript levels were found to be significantly (*P* ≤ 0.05) decreased in the presence of the SINE insertion, but not the SNP, demonstrating that the SINE insertion affects MSTN production at the level of gene transcription. The ~2 Mb genomic region surrounding *MSTN* appears to be highly functionally active in the transcriptional regulation of exercise [48] and therefore, there may be other contributing factors such as long range regulatory elements in this region, that may further influence or modify the phenotype.

Although a similar conclusion was made previously [22,30], our system studied the effects of both polymorphisms—in contrast to exclusively focusing the SINE insertion—thereby validating that the SINE insertion is the relevant functional mutation and not the SNP. It is evident from our data that the sequence variation at the SNP is not the functional source of the trait, however given that the SNP (g.66493737C>T) and the functionally important SINE insertion are in full concordance here validates the reports of the highly significant association between the SNP and best race distance [17,20,23,24,47] and confirms the usefulness of the SNP as a predictive marker of optimal race distance in the Thoroughbred. The SINE insertion must have been introduced or arose in Thoroughbreds containing the C-allele of the SNP [29,30]. Presumably, this haplotype became more prominent due to heightened popularity for shorter-distance races at the turn of the 20^th^ century.

Furthermore, we have for the first time, to our knowledge, provided mechanistic evidence to explain the decreased myostatin production in the presence of the SINE insertion. We have shown that the presence of the SINE insertion results in a repositioning of the *MSTN* transcriptional start site, resulting in initiation within the SINE insertion sequence. Consequently, a larger transcript sequence is produced. This was evidenced by *in silico* predictions and confirmed by experimental TSS mapping. Disruption of the normal TSS can account for the decrease in *MSTN* gene transcription and resultant protein production that occurs in the presence of the SINE insertion.

## Supporting information

S1 Fig The SINE insertion significantly affects myostatin gene expression and protein levels in differentiated C_2_C_12_ cells. Differentiated C_2_C_12_ cells were transfected with MSTN-luciferase plasmids, 24 hours later the media was refreshed to remove the plasmid and lipofectamine mixture. Samples of cultured media were removed at various time points and luciferase assays were performed to measure the activity in these samples and thus the amount of myostatin being produced. Activity data is normalised to the 1 hour time point. (A) *Gaussia* luciferase production as measured by luciferase assay, mean ± SEM of *n* = 3 independent experiments, performed in triplicate. *P* values where shown indicate significance, asterisk above time points indicates variance between groups, asterisk to the right of the line graph indicates variance due to time, for that group. (A-insert) Equivalent data in bar chart format. (B) Luciferase assay data at 72 hours. (C) Gene expression of *MSTN-luciferase* transfected differentiated cells C_2_C_12_ cells at 72 hours, *n* = 4 for each plasmid, performed in at least duplicate. Gene expression was normalised to the expression of *HPRT* (* = *P* ≤ 0.05, ** = *P* ≤ 0.01, *** = *P* ≤ 0.001).
(TIF)

S2 Fig Isolation of *MSTN* DNA and cloning into pBluescript plasmid. DNA was isolated form skeletal muscle tissue as described in materials and methods section. The *MSTN* gene along with extensive promoter region was amplified from this DNA. (A) Shows the PCR amplified *MSTN* DNA (8359 bp) along with restriction enzyme digestions of the same amplified DNA; M1: λ-hindIII digest markers (100 ng); 1: undigested *MSTN* DNA; 2: BsaI digested *MSTN* DNA; 3: BbsI digested *MSTN* DNA; 4: XhoI digested *MSTN* DNA; 5: KpnI digested *MSTN* DNA. Product sizes: BsaI = 5979, 2380; BbsI = 3310, 2826, 1174, 1049; XhoI = 8355, 4*; and KpnI = 8355, 4*. DNA was digested at 37°C for 1 hour 45 minutes and samples were electrophoresed on a 0.8% agarose gel. (B) The *MSTN* DNA was cloned into a pBluescript plasmid by ligation, clones were tested for positive ligation by performing a PCR for the SINE insertion and running the products on a 2% agarose gel alongside GeneRuler DNA marker, thus identifying clones that contained the *MSTN* DNA sequence. (C) DNA was isolated from a bacterial culture of selected positive clones and the DNA was sequenced using M13uni (5'-TGT AAA ACG ACG GCC AGT-3' (forward)) and M13rev (5'-CAG GAA ACA GCT ATG ACC-3' (reverse)) primers by MWG eurofins. Shown here is a snapshot of sequencing data (at ligation point) of clone 6 (shown in red box in (B)) which confirms identification of positive clones based on the presence of the *MSTN* sequence in pBluescript plasmid DNA. (D) Restriction enzyme digests were performed using the pBluescript+MSTN plasmid DNA as an additional confirmation of positive cloning. M1: λ-hindIII digest markers (100 ng); 1: XhoI digest; 2: KpnI digest; 3: XhoI+KpnI digest; 4: SpeI digest; 5: EcoRI digest; 6: PvuII digest. Product sizes: XhoI = 11297 bp; KpnI = 11297 bp; XhoI+KpnI = 8347 bp and 2950 bp; SpeI = 8443 bp and 2584 bp; EcoRI = 5754 bp, 5472 bp and 71 bp*; PvuII = 5783 bp, 2820 bp, 2517 bp and 177 bp*. DNA was digested at 37°C for 1 hour 30 minutes and samples were electrophoresed on a 0.8% agarose gel. * Some small bands are not visible on this gel percentage.
(TIF)

S3 Fig Verification of site-directed mutagenesis modification of *Gaussia* luciferase plasmid and cloning of *MSTN* fragment into *Gaussia* luciferase plasmid. (A) Site-directed mutagenesis (SDM) was employed to alter the *Gaussia* luciferase to add a KpnI site. After SDM the altered plasmid was transformed into competent cells and was selectively grown in culture, DNA was isolated and a sample was sequenced using the following primer; 5'-GGG GTT CCG CGC ACA TTT CCC CG-3', by MWG eurofins. The diagram shows a snapshot of sequencing data (at Site-directed mutagenesis site) of value-read sequencing which was used to confirm correct alteration. (B) pBluescript+MSTN plasmid was digested with XhoI and KpnI to remove the *MSTN* fragment and this was gel purified. The gel purified *MSTN* DNA was then cloned into the modified *Gaussia* luciferase plasmid by ligation. Clones were tested for positive ligation by performing a PCR for the SINE insertion 227 bp polymorphism sequence and running the products on a 2% agarose gel alongside GeneRuler DNA marker, thus identifying clones that contained the *MSTN* DNA sequence. (C) DNA was isolated from an overnight culture of selected positive clones and the DNA was sequenced using using the following primer; 5'-GGG GTT CCG CGC ACA TTT CCC CG-3', by MWG eurofins. Shown here is a snapshot of the sequencing data (at ligation point) of clone 8 (shown in red box in (B)) which confirms identification of positive clones based on the presence of the *MSTN* sequence in *Gaussia* luciferase plasmid DNA. (D) Restriction enzyme digests were performed using the MSTN-luciferase plasmid DNA as an additional confirmation of positive cloning. M1: λ-hindIII digest markers (100 ng); M2: wide range MW markers (Sigma); 1: XhoI digest; 2: KpnI digest; 3: XhoI+KpnI digest; 4: SpeI digest; 5: EcoRI digest; 6: PvuII digest. Product sizes: XhoI = 13219 bp; KpnI = 13219 bp; XhoI+KpnI = 8347 bp and 4872 bp; SpeI = 13219 bp; EcoRI = 7697 bp, 5472 bp and 50 bp*; PvuII = 7499 bp, 3377 bp, 1097 bp, 1069 bp and 177 bp*. DNA was digested at 37°C for 1 hour 30 minutes and samples were electrophoresed on 0.8% agarose gel. * Some small bands are not visible on this gel percentage.
(TIF)

S4 Fig Removal of the *MSTN* SINE insertion 227 bp polymorphism sequence. (A) Shows sequence of *MSTN* SINE insertion and surrounding nucleotides, sequence data obtained from GenScript sequencing of MSTN-luciferase plasmid. (B) GenScript removed the *MSTN* SINE Insertion 227 bp polymorphism sequence by firstly removing the region along with some surrounding nucleotides by digestion with two rare enzymes which cut only once within the entire plasmid and *MSTN* sequence, on either side of the SINE insertion, the fragment was then amplified using GenScripts CloneEz method to obtain the desired new fragment without the 227 bp SINE insertion sequence. The new fragment was cloned back into the plasmid and it was sequenced, thus providing the sequence data shown. (C) Shows a snapshot of the sequencing data showing that the SINE insertion sequence is not present in the final plasmid. Sequence data from before and after the SINE insertion removal was analysed to confirm no other alterations were made to the construct.
(TIF)

S5 Fig Verification of site-directed mutagenesis modification of MSTN-luciferase plasmid to alter SNP g.66493737. A single base change was made by site-directed mutagenesis (SDM) to change the base at SNP g.66493737 from G to A (C to T on negative strand). This alteration was made to two plasmids, the one containing the SINE insertion and the one with the SINE insertion removed. After SDM the altered plasmids were transformed into competent cells and were selectively grown in culture, DNA was isolated and a sample was sequenced using the following primer; 5'-GGG AGA CAG ACA CCT TCA CAG AG-3', by MWG eurofins. (A) Shows a snapshot of MSTN-luciferase plasmid (with SINE insertion) sequencing data (at site-directed mutagenesis site) of value-read sequencing which was used to confirm correct alteration. (B) Shows a snapshot of MSTN-luciferase plasmid (without SINE insertion) sequencing data (at site-directed mutagenesis site) of value-read sequencing which was used to confirm correct alteration.
(TIF)
