# Supplementary figures and images for: The “speed gene” effect of myostatin arises in Thoroughbred horses due to a promoter proximal SINE insertion
Source: PLoS One. 2018 Oct 31;13(10):e0205664. doi: 10.1371/journal.pone.0205664 (PMC6209199; doi:10.1371/journal.pone.0205664)

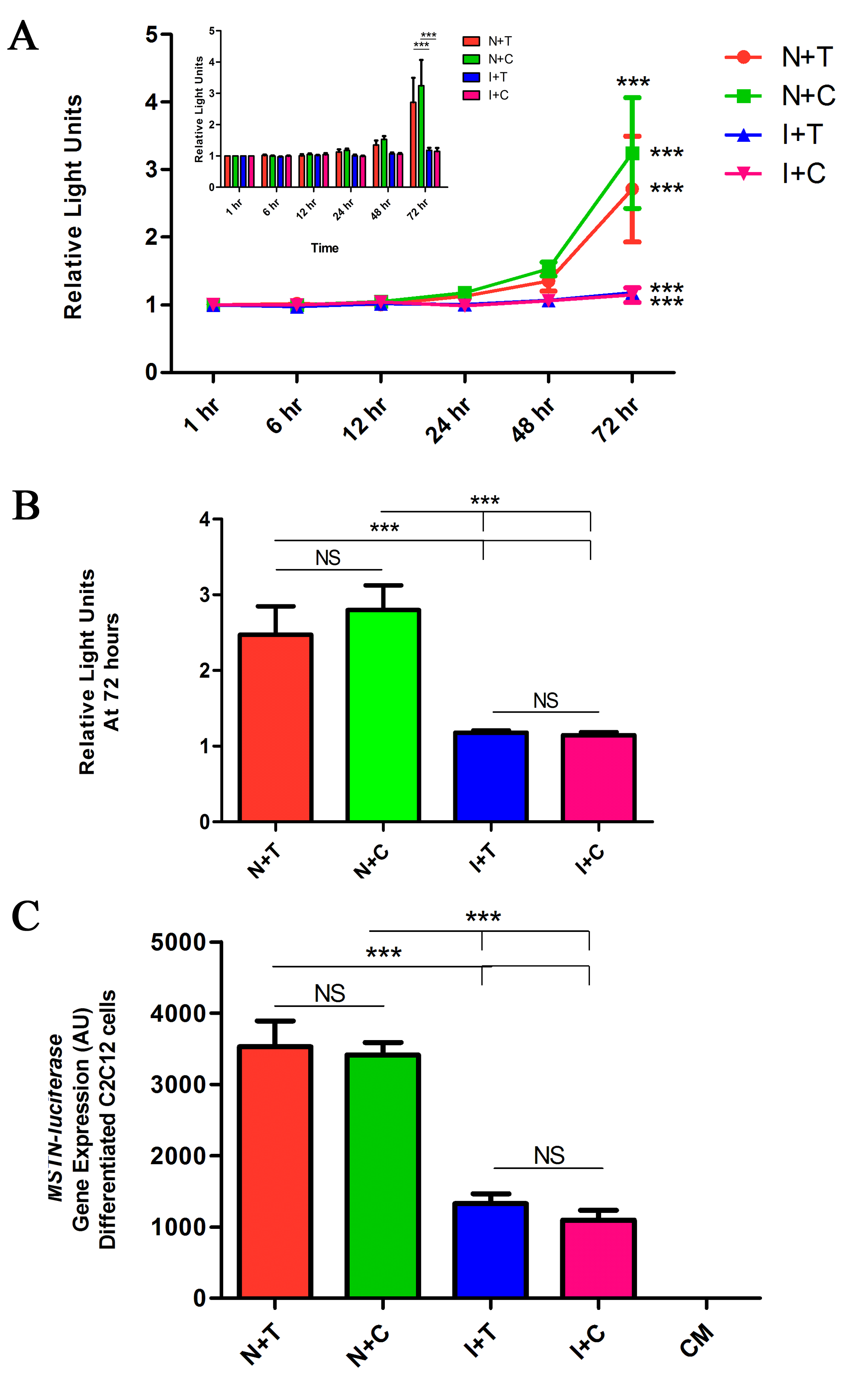

Supplement: S1 Fig — Differentiated C2C12 cells were transfected with MSTN-luciferase plasmids, 24 hours later the media was refreshed to remove the plasmid and lipofectamine mixture. Samples of cultured media were removed at various time points and luciferase assays were performed to measure the activity in these samples and thus the amount of myostatin being produced. Activity data is normalised to the 1 hour time point. (A) Gaussia luciferase production as measured by luciferase assay, mean ± SEM of n = 3 independent experiments, performed in triplicate. P values where shown indicate significance, asterisk above time points indicates variance between groups, asterisk to the right of the line graph indicates variance due to time, for that group. (A-insert) Equivalent data in bar chart format. (B) Luciferase assay data at 72 hours. (C) Gene expression of MSTN-luciferase transfected differentiated cells C2C12 cells at 72 hours, n = 4 for each plasmid, performed in at least duplicate. Gene expression was normalised to the expression of HPRT (* = P ≤ 0.05, ** = P ≤ 0.01, *** = P ≤ 0.001). (TIF) [file pone.0205664.s001.tif]

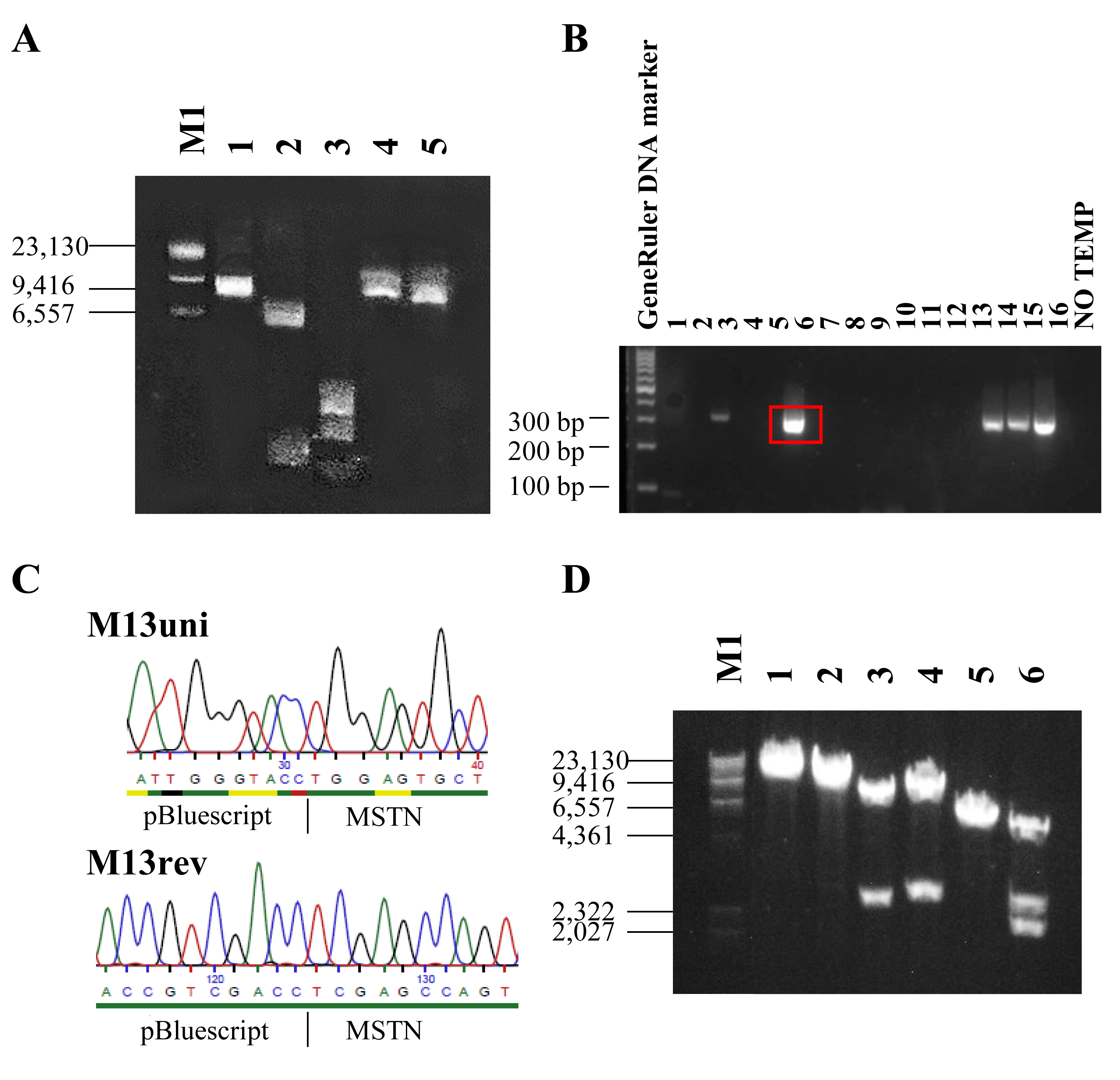

Supplement: S2 Fig — DNA was isolated form skeletal muscle tissue as described in materials and methods section. The MSTN gene along with extensive promoter region was amplified from this DNA. (A) Shows the PCR amplified MSTN DNA (8359 bp) along with restriction enzyme digestions of the same amplified DNA; M1: λ-hindIII digest markers (100 ng); 1: undigested MSTN DNA; 2: BsaI digested MSTN DNA; 3: BbsI digested MSTN DNA; 4: XhoI digested MSTN DNA; 5: KpnI digested MSTN DNA. Product sizes: BsaI = 5979, 2380; BbsI = 3310, 2826, 1174, 1049; XhoI = 8355, 4*; and KpnI = 8355, 4*. DNA was digested at 37°C for 1 hour 45 minutes and samples were electrophoresed on a 0.8% agarose gel. (B) The MSTN DNA was cloned into a pBluescript plasmid by ligation, clones were tested for positive ligation by performing a PCR for the SINE insertion and running the products on a 2% agarose gel alongside GeneRuler DNA marker, thus identifying clones that contained the MSTN DNA sequence. (C) DNA was isolated from a bacterial culture of selected positive clones and the DNA was sequenced using M13uni (5'-TGT AAA ACG ACG GCC AGT-3' (forward)) and M13rev (5'-CAG GAA ACA GCT ATG ACC-3' (reverse)) primers by MWG eurofins. Shown here is a snapshot of sequencing data (at ligation point) of clone 6 (shown in red box in (B)) which confirms identification of positive clones based on the presence of the MSTN sequence in pBluescript plasmid DNA. (D) Restriction enzyme digests were performed using the pBluescript+MSTN plasmid DNA as an additional confirmation of positive cloning. M1: λ-hindIII digest markers (100 ng); 1: XhoI digest; 2: KpnI digest; 3: XhoI+KpnI digest; 4: SpeI digest; 5: EcoRI digest; 6: PvuII digest. Product sizes: XhoI = 11297 bp; KpnI = 11297 bp; XhoI+KpnI = 8347 bp and 2950 bp; SpeI = 8443 bp and 2584 bp; EcoRI = 5754 bp, 5472 bp and 71 bp*; PvuII = 5783 bp, 2820 bp, 2517 bp and 177 bp*. DNA was digested at 37°C for 1 hour 30 minutes and samples were electrophoresed on a 0.8% agarose gel. * Some small ban [file pone.0205664.s002.tif]

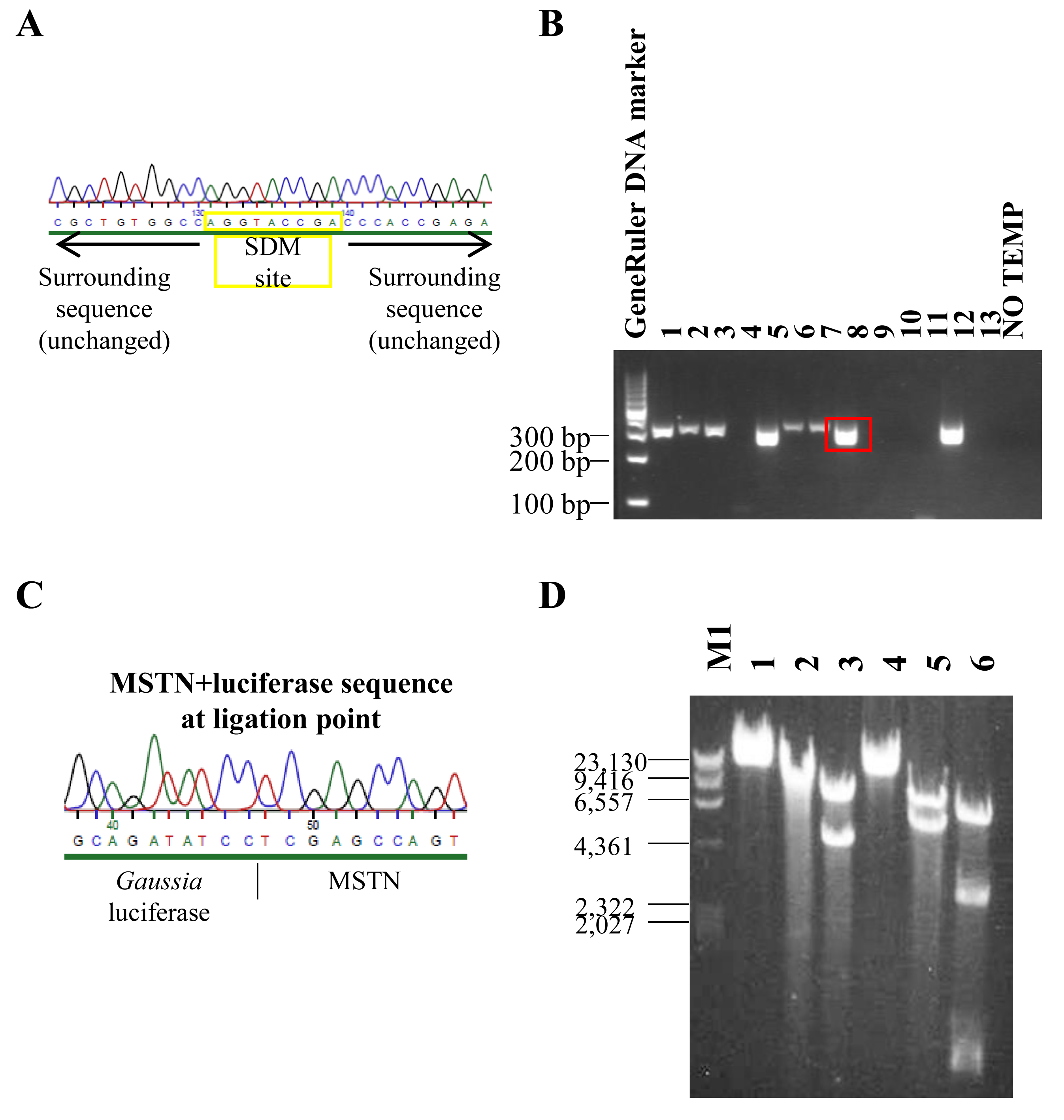

Supplement: S3 Fig — (A) Site-directed mutagenesis (SDM) was employed to alter the Gaussia luciferase to add a KpnI site. After SDM the altered plasmid was transformed into competent cells and was selectively grown in culture, DNA was isolated and a sample was sequenced using the following primer; 5'-GGG GTT CCG CGC ACA TTT CCC CG-3', by MWG eurofins. The diagram shows a snapshot of sequencing data (at Site-directed mutagenesis site) of value-read sequencing which was used to confirm correct alteration. (B) pBluescript+MSTN plasmid was digested with XhoI and KpnI to remove the MSTN fragment and this was gel purified. The gel purified MSTN DNA was then cloned into the modified Gaussia luciferase plasmid by ligation. Clones were tested for positive ligation by performing a PCR for the SINE insertion 227 bp polymorphism sequence and running the products on a 2% agarose gel alongside GeneRuler DNA marker, thus identifying clones that contained the MSTN DNA sequence. (C) DNA was isolated from an overnight culture of selected positive clones and the DNA was sequenced using using the following primer; 5'-GGG GTT CCG CGC ACA TTT CCC CG-3', by MWG eurofins. Shown here is a snapshot of the sequencing data (at ligation point) of clone 8 (shown in red box in (B)) which confirms identification of positive clones based on the presence of the MSTN sequence in Gaussia luciferase plasmid DNA. (D) Restriction enzyme digests were performed using the MSTN-luciferase plasmid DNA as an additional confirmation of positive cloning. M1: λ-hindIII digest markers (100 ng); M2: wide range MW markers (Sigma); 1: XhoI digest; 2: KpnI digest; 3: XhoI+KpnI digest; 4: SpeI digest; 5: EcoRI digest; 6: PvuII digest. Product sizes: XhoI = 13219 bp; KpnI = 13219 bp; XhoI+KpnI = 8347 bp and 4872 bp; SpeI = 13219 bp; EcoRI = 7697 bp, 5472 bp and 50 bp*; PvuII = 7499 bp, 3377 bp, 1097 bp, 1069 bp and 177 bp*. DNA was digested at 37°C for 1 hour 30 minutes and samples were electrophoresed on 0.8% agarose gel. * Some small band [file pone.0205664.s003.tif]

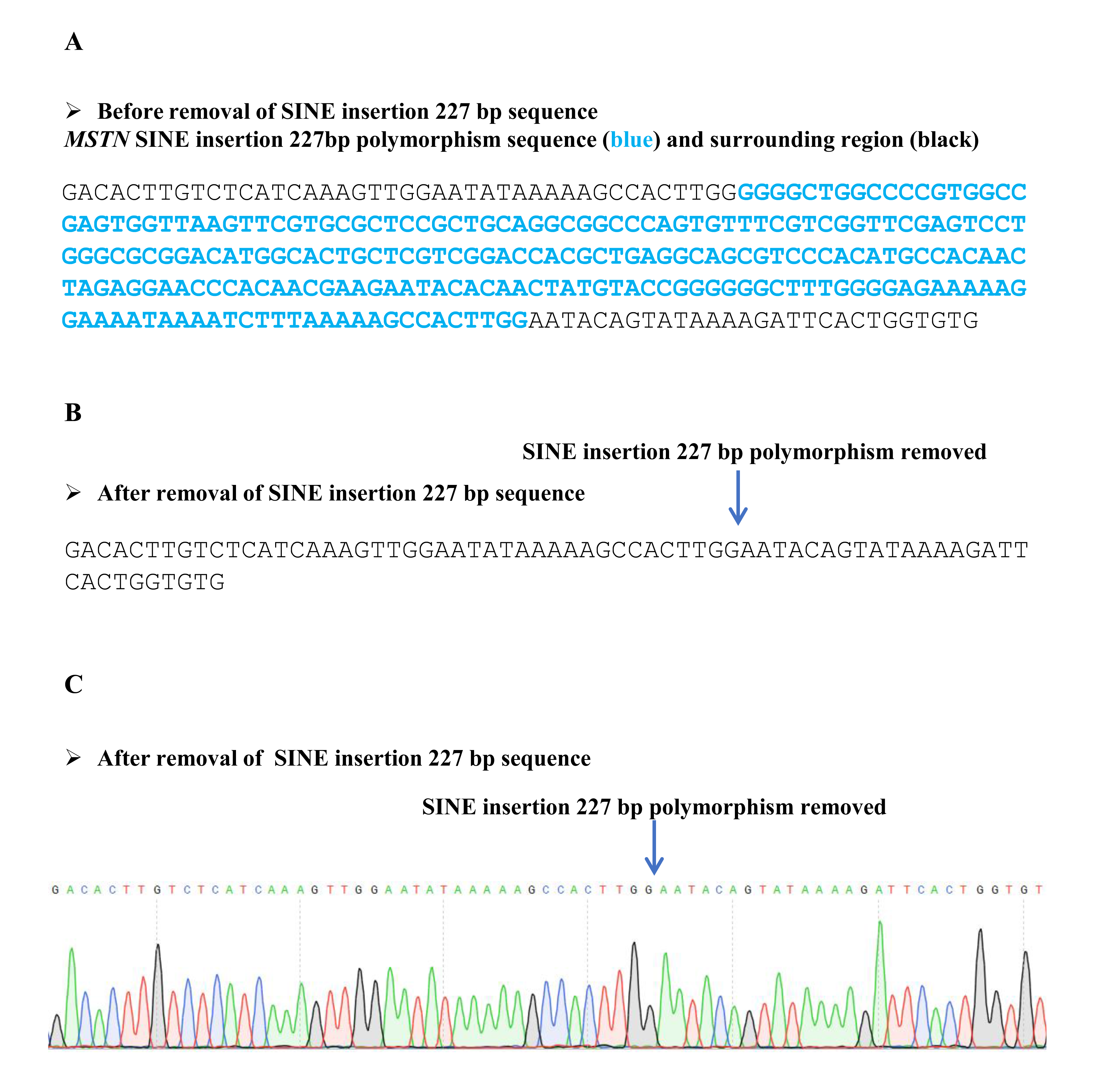

Supplement: S4 Fig — (A) Shows sequence of MSTN SINE insertion and surrounding nucleotides, sequence data obtained from GenScript sequencing of MSTN-luciferase plasmid. (B) GenScript removed the MSTN SINE Insertion 227 bp polymorphism sequence by firstly removing the region along with some surrounding nucleotides by digestion with two rare enzymes which cut only once within the entire plasmid and MSTN sequence, on either side of the SINE insertion, the fragment was then amplified using GenScripts CloneEz method to obtain the desired new fragment without the 227 bp SINE insertion sequence. The new fragment was cloned back into the plasmid and it was sequenced, thus providing the sequence data shown. (C) Shows a snapshot of the sequencing data showing that the SINE insertion sequence is not present in the final plasmid. Sequence data from before and after the SINE insertion removal was analysed to confirm no other alterations were made to the construct. (TIF) [file pone.0205664.s004.tif]

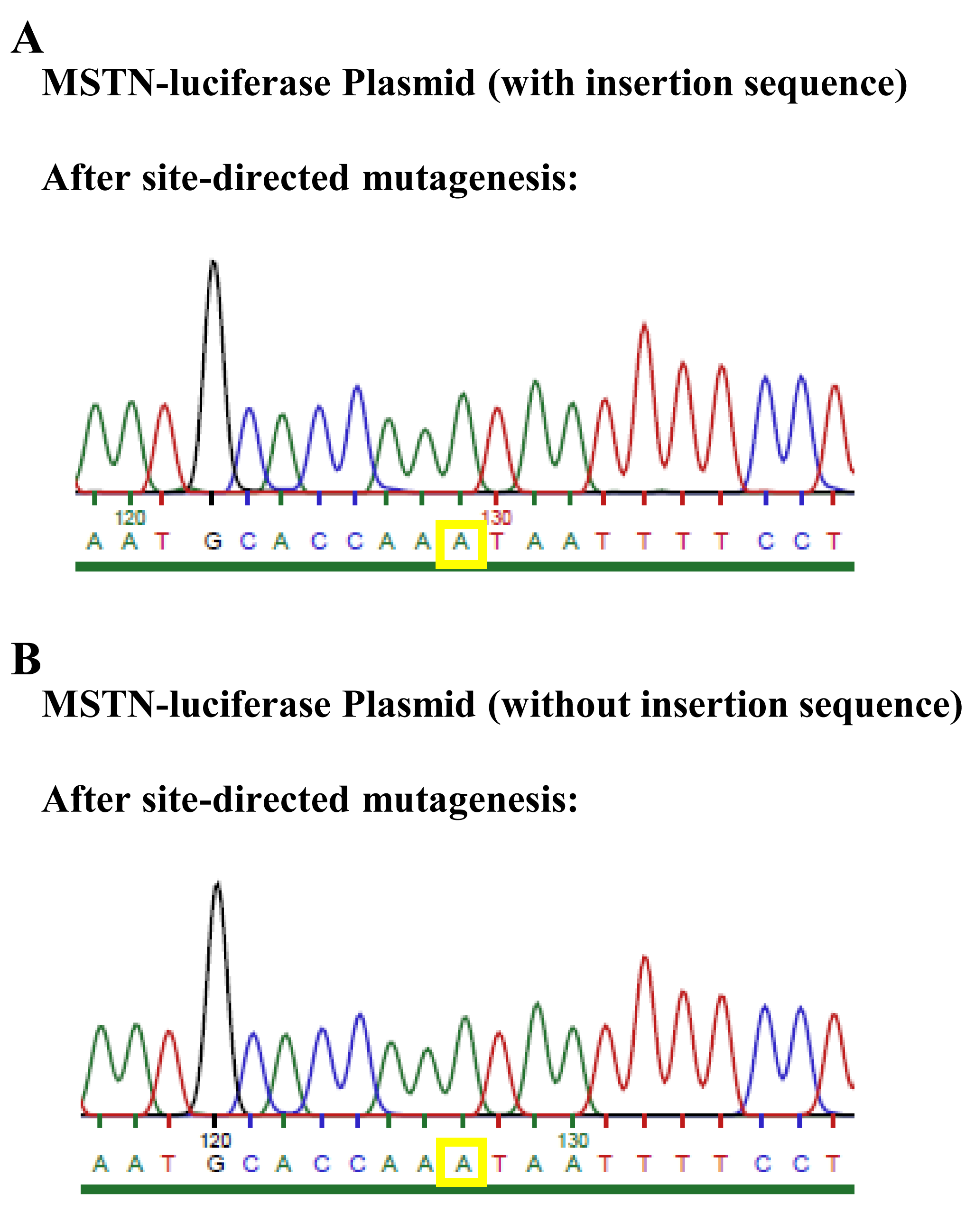

Supplement: S5 Fig — A single base change was made by site-directed mutagenesis (SDM) to change the base at SNP g.66493737 from G to A (C to T on negative strand). This alteration was made to two plasmids, the one containing the SINE insertion and the one with the SINE insertion removed. After SDM the altered plasmids were transformed into competent cells and were selectively grown in culture, DNA was isolated and a sample was sequenced using the following primer; 5'-GGG AGA CAG ACA CCT TCA CAG AG-3', by MWG eurofins. (A) Shows a snapshot of MSTN-luciferase plasmid (with SINE insertion) sequencing data (at site-directed mutagenesis site) of value-read sequencing which was used to confirm correct alteration. (B) Shows a snapshot of MSTN-luciferase plasmid (without SINE insertion) sequencing data (at site-directed mutagenesis site) of value-read sequencing which was used to confirm correct alteration. (TIF) [file pone.0205664.s005.tif]
